# A Follow-Up on Psychiatric Symptoms and Post-Traumatic Stress Disorders in Tuareg Refugees in Burkina Faso

**DOI:** 10.3389/fpsyt.2018.00127

**Published:** 2018-04-24

**Authors:** Mauro Giovanni Carta, Daniela Moro, Fadimata Wallet Oumar, Maria Francesca Moro, Mirra Pintus, Elisa Pintus, Luigi Minerba, Federica Sancassiani, Elisabetta Pascolo-Fabrici, Antonio Preti, Dinesh Kumar Bhugra

**Affiliations:** ^1^Department of Medical Sciences and Public Health, University of Cagliari, Cagliari, Italy; ^2^Corso di Laurea in Tecnica della Riabilitazione Psichiatrica, University of Trieste, Trieste, Italy; ^3^Health Service and Population Research Department (HSPRD), Institute of Psychiatry, King’s College London, London, United Kingdom

**Keywords:** post-traumatic stress disorder, refugees, war traumas, follow-up, screening tools, Tuareg

## Abstract

**Introduction:**

The aim of this study was to carry out a 2-year follow-up of refugees in a camp in Burkina Faso who had been interviewed previously. We also aimed to verify whether the general conditions in which they lived (e.g., protection by international organizations and the conclusion of negotiations and new hope of returning to Mali and reunification with surviving family members) would affect their mental health state.

**Methods:**

This is a cross-sectional study repeated over time on a cohort of refugees. People living in the Subgandé camp who had participated in the first survey in 2012 were identified using informational chains and approached for follow-up. Those who agreed were interviewed using the Short Screening Scale for post-traumatic stress disorder (PTSD) and the K6 scale, French versions, to measure general psychopathology and the level of impairment.

**Results:**

The second survey shows a dramatic decrease in psychopathological symptoms (positivity at K6 scale). Improvement was also conspicuous in the frequency of people with stress symptoms (positivity at Short Screening Scale for PTSD and simultaneous positivity to K6 scale). The frequency of people screened positive at the Short Screening Scale for PTSD had also decreased, but the level of improvement was not pronounced.

**Conclusion:**

Our findings confirm that when physical conditions improve, psychological symptoms can also improve. Although in the studied sample psychological factors, such as the hope of returning to their own land and thus the possibility of maintaining ethnic cohesion, may have played a role, future research carried out with a proper methodology and sufficient resources to identify protective factors is needed.

## Introduction

Populations fleeing war have often suffered or have witnessed violence, persecution, and imprisonment; they are at high risk of exposure to traumas and consequently to manifesting psychopathologies, stress-related disorders, and post-traumatic stress disorder (PTSD) (1). Regarding the condition of refugees, factors inherent in the conditions of escaping, traveling, and reception in the host country can also aggravate the psychopathology linked to previous traumas, or even add new ones (2).

The prevalence of PTSD in refugee camps in Africa varies from 15.8% in Ethiopia to 37.4% in Algeria (3). The presence of any severe mental disorder ranged from 17.5% in Ethiopian to 60.5% in Algerian refugees (3). In people of three Sub-Saharan refugee camps in postwar scenarios in Darfur Hamid and Musa, 54% of people were found positive at a screening test for PTSD and 70% positive in a screening test for general distress (4).

The literature contains several studies on small cohorts in refugee camps suffering from disorders who had been subjected to specific treatments (2, 5). However, studies on population samples or entire refugee populations repeated over time are infrequent. One of the studies, considered as a milestone in this field, shows in Bosnian refugees in Croatia that 45% of those who met the DSM-IV criteria for depression or PTSD presented these disorders 3 years later, and 16% of asymptomatic persons developed the disorders (6, 7). A decrease in psychiatric symptoms at follow-up was shown 10 years later, but people suffering from PTSD showed an upward trend and the presence of PTSD was found associated with the unsuccessful extinction of traumatic memories (8).

The few studies found in the literature do not clarify what social mechanisms may occur in the processes of recovery of hope, also because the different circumstances studied probably present specific variables that characterize each situation (9).

However, the results of some studies, even on minors, appear to suggest that restoring a condition of safety and responding to primary needs is not always associated with an improvement in general and stress-related psychopathology (9, 10).

We had previously evaluated a sample of refugees of Tuareg ethnicity who were living in the Subgandé refugee camp in Burkina Faso (11). At that time (immediately after the crisis in Mali), refugees in the camp were faced with strong tensions and fear (11). There were no international organizations guaranteeing security and survival. These refugees had fled from Mali with very little information on those who had remained behind. Meanwhile, interventions by Islamic extremists meant that the political and military situation was not going well for the Tuaregs, who were fighting for independence. The majority of the Tuareg rebels were in fact engaged in the MLNA (Mouvement de Liberation de l’Azawar), the lay and losing party of the Malian rebels (1).

At that time, around 60% of our sample had screened positive for the contemporary presence of both psychopathological stress-related symptoms (as positivity to Short Screening Scale for PTSD (12)) and for the presence of general psychopathological symptoms and impairment linked to psychopathology [as positivity to the K6 screening scale (13)], thus indicating severe mental distress and probable PTSD. Women aged 40 and older were found to be at higher risk of PTSD symptoms. Younger women (39 or younger) had higher frequencies of K6 positivity, but the distribution of people with both PTSD and K6 scales positivity was homogeneous by gender and age (11).

During the 2-year period, many people had left the Subgandé camp and had been relocated to other camps where they appeared to be better supported. In this follow-up study 2 years later, we retraced a substantial portion of the first sample, but many were about to return to Mali.

The aim of the follow-up is to ascertain the health status of those previously interviewed and those we were able to trace. We wanted to see whether the amended general conditions (e.g., protection provided by international organizations and the conclusion of negotiations) had contributed to changes in the levels of mental distress.

## Materials and Methods

### Design

Cross-sectional study repeated over time on a cohort of refugees. This study compares data collected in a first survey (wave 1) (11), with new data collected in the traced individuals of the same sample using the same tools after 2 years (wave 2).

### Sample and Framework

Data were collected in three refugee camps in Burkina Faso between July and August 2014.

People who were living in the Subgandé camp in August and October 2012 and who had participated in the first survey were traced by the researcher in the field (FWO) (11) using informational chains.

At the follow-up, we were inevitably unable to trace all participants, since many had returned to Mali or moved to Europe or other African countries (such as Morocco, Niger, or Algeria). Some did remain in Burkina Faso but were no longer in the camps and could not be traced.

### Ethics

Written informed consent was obtained from each participant in the study. Each participant was identified with a code number without collecting any data leading to identification of individuals. The ethics committee of the Azienda Mista Ospedaliero Universitaria, Cagliari, Italy approved the study, which had also been approved by the local camp authorities. The research was conducted according to the Helsinki Declaration.

### Instruments and Interviews

Each subject taking part in the study was interviewed in French by one of the researchers (FWO). The assessment instruments were as before: (a) a brief introduction; (b) the Short Screening Scale for DSM-IV Posttraumatic Stress Disorder (PTSD Screening Scale), a screening tool based on seven key symptoms/items with yes/no answers (12). Positivity to a single item is worth 1 point, the total score is given by the sum of the points, i.e., the sum of the key symptoms detected (12). The PTSD Screening Scale also presents a list of the five most significant traumatic events selected by authors of the scale on the basis of epidemiological surveys that revealed such events as the most important and frequent in populations at risk: death of a family member, a family relative away at war, severe issues with food, injury, and issues in housing; furthermore, another open item marked “other cause” requiring specific details (12); (c) the K6 scale for the screening of severe anxiety and mood disorders (13). K6 scale consists of six items/symptoms (as reported in Table 5). For each of the 6 symptoms detected, the K6 scale encodes a score from 1 (all the time) to 6 (none of the time). The sum of the total score is used for the screening, but it is possible to monitor the evolution of each symptom over time based on the specific score. With additional items, the K6 scale also determines the mean number of contacts with professionals (physicians, psychologists, nurses) for counseling and the days of disability due to psychosocial factors at waves 1 and 2.

The French version of such instruments had previously been validated (14) and adapted for West Africa French (15). Prior to the wave 1 study, we had also conducted a pilot study on a small sample of Tuareg refugees in Europe aimed to measure the accuracy of these instruments in comparison with a gold standard, i.e., a diagnosis made by a trained psychiatrist by means of a structured interview. As previously described in this specific sample (11), the best cutoff scores for the PTSD screening scale and for the K6 scale were established, respectively, at a score of 4 (sensitivity = 0.66 and specificity = 0.55) and at the score of 13 (sensitivity = 0.75 and specificity = 0.59).

Thus, in this wave 2 study, we adopted the same cutoff for both tools (PTSD and K6 scales) used in wave 1, according to the preliminary survey: 4 for PTSD screening scale and 13 for K6 scale (11).

As K6 scale measures the most general and severe anxiety and depressive symptoms and the level of impairment due to the psychopathology, positivity both at PTSD and K6 scales identifies the most severe cases of PTSD.

### Statistical Analysis

The point prevalence rates of PTSD with and without positiveness at K6 scale were calculated for gender and age [≤39 and >39 in accordance with the previous wave survey (11)]. Statistical significance was calculated using the χ2 test in 2 × 2 tables.

Comparison of the size of the decrease (wave 1 vs wave 2) between the frequencies of PTSD screening scale positivity and the amount of the decrease in the frequencies of K6 scale positivity and of both K6 and PTSD scales positivity were calculated by means of the Cochran–Armitage chi square test for trend (16).

The odds ratio 95% confidence intervals (OR 95% CI) were calculated using Miettinen’s simplified method (17). The numeric interval variables were compared by means of ANOVA one-way statistics.

The evolution of each symptom detected by K6 scale over time, wave 1 vs wave 2, based on the specific score, was also measured. A multivariate logistic regression analysis was carried out to measure the simultaneous effect of different independent variables on PTSD screening scale positivity as well as K6 scale positivity at wave 2. The following independent variables were considered in the analysis: age (dichotomy >39 vs ≤39), gender, death of a family member, issues with food, relative away in war, physical damages, and problems with housing. The logistic regression analysis was conducted following the backwards procedure, fitting all variables, and sequentially discarding non-significant values.

## Results

A total of 207 people were traced and 190 (91.79%) agreed to the interview; they represented 46.45% of the wave 1 sample (*N* = 409). Table 1 shows the socio-demographic characteristics of the sample as well as the risk factors; the number of contacts for counseling with professionals and the days of disability due to psychosocial factors in the last 30 days at waves 1 and 2.

**Table 1:** Sample, risk factors, use of counseling, and days of disability due to psychosocial factors in waves 1 and 2.

	Wave I, *N*(%), **Mean ± SD	Wave II	Chi square 1df, *ANOVA 1 way F, 1,597,598 DF	*P*	OR	CI 95%
Female	230 (56.2%)	125 (65.8)	4.906	0.027	0.67	0.46–0.97
≤39 years old	249 (60.9%)	91 (47,9)	8.913	0.003	1.69	1.07–1.52
>5 years education	32 (7.8%)	13 (6.8%)	0.824	0.364	1.35	0.67–2.65
Married	251 (61.4%)	102 (53.7)	3.166	0.075	1.37	0.95–1.97
More than three children	158 (38.3)	69 (36.3%)	0.295	0.587	1.10	0.76–1.60
Death of a family member	333 (81.4)	172 (90.5)	8.013	0.004	0.46	0.25–0.14
Family members far away because of the war	384 (93.9%)	182 (95.8%)	0.902	0.342	0.67	0.27–1.60
Severe problems with food	375 (91.7%)	32 (16.8%)	333.68***	<0.0001	54.4	31.5–94.8
Injury or physical damage to self or acquaintances	338 (82.6)	170 (89.5)	4.701	0.030	0.56	0.32–0.98
Poor housing	385 (94.1%)	11 (5.8%)	451.9***	<0.0001	261.0	118.9–588.0
Contacts in the last 30 days with professionals for psychosocial disabilities*	9.91 ± 13.03**	6.93 ± 9.49**	*F = 7.971	0.005		
Totally impaired in the last 30 days due to psychosocial disabilities*	11.19 ± 12.04**	7.54 ± 9.76**	*F = 13.503***	<0.0001		

*p < 0.05; **p < 0.01; ***p < 0.001.

Females were more represented in percentage at wave 2 in comparison with wave 1 (65.8 vs 56.2% chi square 1 DF 4.906, *P* = 0.027); younger females (below 39 years) were 47.9% against 60.9% in the sample of 2 years before (chi square 1 DF = 8.913, *P* = 0.003). The other demographic variables (marital status, presence of more than three children, frequency of people with more than 5 years of education) showed no differences between the two samples in waves 1 and 2.

Regarding the specific risk factors: “Family members far away because of the war” showed no differences in the samples at waves 1 and 2; “Death of a family member” (81.4 vs 90.5%, *P* = 0.0004) and “Injury or physical damage to self or others known to the individual” (82.6 vs 89.5%, *P* = 0.030) showed slightly higher frequencies in the wave 2 sample. On the contrary, “Severe problems with food” (91.7 vs 16.8%, *P* < 0.0001), and “Poor housing” (94.5 vs. 5.8%, *P* < 0.0001) showed a clear improvement at wave 2.

The days of disability due to mood or anxiety problems in the 30 days before the interview significantly decreased at wave 2 (11.19 ± 12.04 vs 7.54 ± 9.76, *P* < 0.0001). Thus, the number of times that a specialist (doctor, psychologist, or nurse) was consulted for such problems also decreased (9.91 ± 13.03 vs 6.93 ± 9.49; *P* = 0.005).

Table 2 compares the point prevalence of people screened positive for PTSD symptoms in the first survey and after 2 years.

**Table 2:** People screened positive for post-traumatic stress disorder (PTSD) symptoms in the first survey and after 2 years.

	Wave I PTSD	Wave II PTSD	Chi square	*P*	OR CI 95%
Males	152 (85.4%)	48 (73.84%)	3.95	<0.042	1.99; 0.95–4.18
Females	198 (86.1%)	80 (66.70%)	23.26	<0.0001	3.48; 2.00–6.07
≤39	202 (81.1%)	52 (57.1%)	20.28	<0.0001	3.22; 1.85–5.62
>39	148 (93.1%)	76 (76.8%)	5.68	<0.017	2.65; 1.09–24.93

Total	350 (85.6%)	128 (67.4%)	26.67	<0.0001	2.87; 1.87–4.42

The differences in the prevalence of PTSD between the two waves achieve statistical significance in the total sample (OR 2.87, CI 95% 1.87–4.42, Chi square 26.67, *P* < 0.0001). The frequency decreased somewhat less in male (difference wave II vs wave I, OR male 1.99, CI 95% 0.95–4.18, Chi square 3.95, *P* < 0.042 vs OR female 3.48, CI 95% 2.00–6.07, Chi square 23.26, *P* < 0.0001) and in older people (difference wave II vs wave I, OR >39 years old 2.65, CI 95% 1.09–24.93, Chi square 5.68, *P* < 0.017 vs OR 16–39 years old 3.32, CI 95% 1.85–5.62, Chi square 20.28, *P* < 0.0001).

A similar trend, but even more marked, is also highlighted in people screened positive for anxiety and mood disorders by K6 scale. In fact, the positives at the PTSD screening scale were 85.6% at wave 1 and 67.4% at wave 2 (see Table 2). By contrast, the positives at K6 scale were 75.00% at wave 1 and 15.8% at wave 2 (Table 3). Thus, the decrease from wave 1 to wave 2 was 18.2% for PTSD screening scale and 59.2% for K6 scale (difference of the trends Chi square 40.00, 1 GL, *P* < 0.00001).

**Table 3:** People screened positive for mood and anxiety disorders in the first survey and after 2 years (K6 positives).

	Wave I K6 positives	Wave II K6 positives	Chi square	*P*	OR CI 95%
Males	142 (79.8%)	11 (16.9%)	79.41	<0.0001	18.84; 8.50–42.61
Females	164 (71.3%)	19 (15.2%)	102.01	<0.0001	13.86; 7.62–25.47
≤39	202 (81.1%)	17 (18.7%)	115.51	<0.0001	19.44; 10.07–37.97
>39	104 (65.4%)	13 (13.1%)	60.75	<0.0001	11.34; 5.55–23.59

Total	306 (75.0%)	30 (15.8%)	189.73	<0.0001	16.65; 10.37–26.84

The positives at both PTSD and K6 scales were 61.8% at wave 1 and 9.5% at wave 2 (Table 4). As reported above, the positives at the PTSD screening scale alone were 85.6% at wave 1 and 67.4% at wave 2 (see Table 2). The decrease from wave 1 to wave 2 was 18.2% for PTSD screening scale alone and 52.3% for positivity for both K6 and PTSD scales (difference of the trends Chi square 44.26, 1 GL, *P* < 0.00001).

**Table 4:** People screened positive for both post-traumatic stress disorder (PTSD) symptoms and anxiety and mood disorders in the first survey and after 2 years (positivity to both PTSD screening scale and K6).

	Wave I K6 + PTSD	Wave II K6 + PTSD	Chi square	*P*	OR CI 95%
Males	119 (66.9%)	8 (12.3%)	17.44	<0.0001	5.55; 2.18–14.51
Females	133 (57.8%)	10 (6.25%)	108.08	<0.0001	20.57; 8.83–51.55
≤39	158 (63.5%)	7 (7.6%)	82.96	<0.0001	20.83; 21.27–130.83
>39	94 (59.1%)	11 (11.1%)	58.26	<0.0001	11.57; 5.48–24.93

Total	252 (61.8%)	18 (9.5%)	143.13	<0.0001	15.43; 8.90–27.07

Table 5 shows the evolution of specific symptoms detected by K6 scale at waves 1 and 2. Four symptoms show a marked improvement at wave 2 with a statistically significant difference from wave 1 in feeling: “nervous” (*P* < 0.0001); “hopeless” (*P* < 0.0001); “that everything is an effort” (*P* < 0.0001); “restless or fidgety” (*P* = 0.026) (*P* < 0.0001), as well as the total of the K6 score (*P* < 0.002). Two symptoms showed no statistically significant differences in feeling “so depressed that you could get nothing” and “worthless,” with the latter symptom showing an inverse trend with a tendency to a lower score at wave 2.

**Table 5:** Presence of specific symptoms detected by K6 at waves 1 and 2 (ANOVA 1 way with Bonferroni correction).

	Wave 1 (Mean ± SD)	Wave 2 (Mean ± SD)	*F*1,597,598 df	*P*
K6. Item 1 = nervous	2.54 ± 1.23	3.06 ± 1.14	24.270	<0.0001
K6. Item 2 = hopeless	2.81 ± 1.21	3.24 ± 1.18	16.642	<0.0001
K6. Item 3 = restless or fidgety	3.24 ± 1.12	3.47 ± 1.28	4.988	0.026
K6. Item 4 = so depressed that nothing could cheer you up	3.09 ± 1.28	3.20 ± 1.13	1.030	0.311
K6. Item 5 = that everything was an effort	2.66 ± 1.34	3.20 ± 1.12	23.291	<0.0001
K6. Item 6 = worthless	3.43 ± 1.51	3.19 1.21	3.696	0.055

Total score	17.77 ± 5.12	19.20 ± 5.75	9.347	0.002

Table 6 shows the analysis of determinants associated with positivity to the PTSD screening scale revealed by multivariate logistic regression analysis. No factor, between gender and those reported in the PTSD screening scale, was shown to be specifically associated with PTSD except young (≤ 9) age, which emerges as a protective factor at a statistically significant level: (coefficient = −0.860, Robust SE = 0.337, CI 95% −1.521 to −0.199) (Table 6).

**Table 6:** Logit regression—dependent variable: positive to post-traumatic stress disorder (PTSD) screening scale (cut-off score ≥4).

Dependent variable: positive to PTSD (≥4)	Coef.	Robust standard error	95% Interval confidence	
Age (≤39 years)	−0.860**	0.337	−1.521	−0.199
Gender (male)	−0.355	0.397	−1.133	0.422
Death of a family member	−1.707	1.942	−5.514	2.100
Severe problems with food	0.808	0.547	−0.263	1.880
Injury or physical damage to self or acquaintances	2.286*	1.261	−0.187	4.758
Poor housing	1.548*	0.887	−0.191	3.287
N. obs.	190			
Pseudo R-square	0.093			

* and ** are significance level at 10, and 5%.

Table 7 shows the analysis of determinants associated with positivity to the K6 scale performed by multivariate logistic regression analysis. Of those in the PTSD screening scale, no factors, including gender and age, were shown to be specifically associated with a statistically significant level for people with positivity to K6 scale (Table 7).

**Table 7:** Logit regression—dependent variable: positive to post-traumatic stress disorder (PTSD) screening scale (cutoff score ≥4) and K6 (cutoff score ≥13).

	Coef.	Robust standard error	95% Interval confidence	
Age (≤ 39 years)	−0.600*	0.310	−1.208	0.007
Gender (male)	−0.043	0.363	−0.755	0.669
Death of a family member	0.088	1.643	−3.133	3.309
Severe problems with food	0.712	0.503	−0.274	1.697
Injury or physical damage to self or acquaintances	1.029	0.898	−0.730	2.789
Poor housing	0.446	0.786	−1.094	1.986
N. obs.	190			
Pseudo R-square	0.041			

* is significance level at 10%.

## Discussion

The present work was conducted from July to August 2014 with the aim of checking the status of mental health of people we were able to trace in the refugee camps of Burkina Faso who had been involved in a previous survey.

The sample we analyzed in this second study has proportionally fewer male and younger subjects. This may be because those who had already left were more likely to be male and younger. Furthermore, less than half of the original sample completed the second survey, so it is theoretically possible that those who were more seriously ill may have been placed elsewhere.

It is apparent that the second wave shows a dramatic decrease in psychopathological symptoms (K6 scale positivity). The improvement was also conspicuous regarding the frequency of people with both stress symptoms (positivity at the PTSD Screening Scale) and simultaneous positivity to K6 scale, a tool that, as previously indicated, measures the presence of symptoms of general psychopathology but also the level of impairment. The number of participants reporting symptoms of PTSD dropped considerably, although the improvement was less pronounced in comparison with the other two parameters considered (people with K6 scale positivity measuring general psychopathology and impairment and people with both PTSD and K6 scales positivity). This change in these parameters is much higher than expected based on the literature concerning studies repeated over time (2, 6–8, 10) on refugees coming from wars. However, the fact that PTSD symptoms improve with more difficulty than other anxious or depressive ones is a fact confirmed by other studies (17).

Improvement in the general conditions of subsistence in wave 2 is well evidenced by the fact that almost all the sample examined at wave 1 complained about the conditions of “severe problems with food” and “poor housing,” while on the contrary at wave 2 almost the entire sample reported being satisfied.

By contrast, a larger number of people at wave 2 lamented “death of a family member” and “injury or physical damage to self or others known to the individual.” The difference may be due to the fact that after 2 years of war a greater number of people had been killed and that at the time the war was over and contacts with homes reestablished, many had news of relatives’ deaths, of which they were previously unaware. It must also be considered that since these differences are not marked, the fact that only half of the wave 1 sample was re-interviewed, the difference may be due to a selection bias.

However, one element of great interest is that while the death of a family member at wave 1 was associated with a higher risk of positivity in the PTSD screening scale by the logistic regression analysis (11), this association is not present at wave 2 using the same statistical method. Even the absence of food, although much diminished as a frequency, is no longer associated with risk of PTSD at wave 2, while it is associated with risk at wave 1 (11). Furthermore, old people were statistically less likely to be positive on the K6 scale at wave 1 (11), and this factor was no longer associated with the K6 scale positivity at wave 2. These elements suggest the presence of new protection factors at wave 2.

There is no doubt that the effects of war tend to impair mental health in the long term in several ways. Community surveys have shown specifically an association between the exposure to wars and PTSD-related psychopathology. Mollica et al., on a sample of Bosnian refugees after 3 years from the war, found nearly half the people presenting significant symptoms of PTSD (7); a follow-up carried out 10 years after the Bosnian war by the same research group (8) still showed severe consequences on people exposed to traumatic events. Intriguingly, in a Lebanese community sample, Karam et al. (18) found a higher risk of psychiatric disorders even 20 years after the war. Those who were children during the war showed a higher risk. More than half of the Israeli soldiers who took part in operations in the same war in Lebanon reported triple comorbidity (PTSD + anxiety + depression) during their lifetimes (19), and almost 20% suffered from late onset PTSD (20).

The specific framework and related socio-cultural risk factors have a clear impact on the outcome and can determine specific profiles of risk in each group (21–26).

Our first wave study that explored the risk of trauma and stressor-related disorders in the Tuareg sample of refugees in the Subgandé camp in Burkina Faso was conducted immediately after the crisis had occurred, when hundreds of thousands of Malian Tuareg were fleeing to poor neighboring countries as refugees (11). In that study, around 60% of the participants fulfilled the criteria for trauma- and stressor-related disorders and were also found positive at the K6 scale (11).

The remarkably higher frequency of mental suffering, even more in comparison with similar studies, was hypothesized as being associated with the terrible hardships that those people were facing. At that time, the refugee camps were receiving virtually no international aid and the camp lacked food and water.

The situation changed considerably in the following months before the second study was carried out. Since the end of 2012, the main refugee camps in Burkina Faso, as well as in other neighboring countries, such as Mauritania, Niger, and Algeria, are under the protection of the High Commissioner of the United Nations and receive humanitarian aid as well as medical support (27). The Subgandé camp was closed in 2014, and refugees from this camp were moved to other, more organized camps.

Although each specific postwar refugee condition is peculiar, based on the literature (9, 10), it is unlikely that such a sharp improvement in psychopathological conditions can be hypothesized as a consequence of the sole improvement in the general conditions of board and lodging. To consider is that the best psychopathological conditions at wave 2 were also accompanied by a lowering of the days of disability in the 30 days before the interview and by the decreasing of number of consultations for psychopathological problems. From this standpoint, we must also take into account that at wave 2 many more professionals were available and, therefore, the decrease in consultations should be noted as relevant.

It is, therefore, possible that the change in the political context that provided a hope of survival as individuals, as a group and a concrete possibility of returning home, may have played a role in the psychological wellbeing of the sample.

In fact, the government of Mali signed a preliminary agreement in Ouagadougou in June 2013 with the northern rebel groups, including the Mouvement National de Libération de l’Azawad (MNLA) to which the majority of the Tuaregs adheres, for a peace treaty. The peace talks after a period of uncertainty regained momentum in Algiers in 2014 (1).

What can be hypothesized is that at the time of the second survey, the Tuareg refugees in Burkina Faso had begun to hope for the possibility of a future. The stressful conditions were in fact the same, apart from somewhat better food and living conditions. Therefore, it is interesting to note that the PTSD scores decreased albeit not to the dramatic extent shown by the positivity in general psychopathology and impairment (K6 scale positivity), or of those having both general psychopathologic symptoms with impairment and symptoms of PTSD.

### Limitations

The study has several major limitations. First, fewer than half of the original participants were traced; second, psychiatric diagnoses, such as PTSD in other cultures, may have limited validity and these tools are quite limited in capturing the almost ubiquitous suffering in populations still available (28), and that, therefore, this methodology has intrinsic limitations.

Another substantial limitation derives from the fact that it is an observational study conducted without a control group. Indeed, the main problem of this study is the absence of a comparison group: one cannot assert that the improvement is related to improved living conditions and/or the restoring of hope with the support of a randomized controlled design.

It is, however, understandable that in this type of research describing the historical evolution of a human phenomenon an experimental design is impossible owing to logistical and ethical problems: (1) given the conditions in which the study was conducted in wave 1, when there was no international support organization, it would have been impossible to conduct a study with an experimental design or, in any case, with more refined tools of evaluation; (2) it would be unethical not to provide all people with the same type of food and logistical support.

The authors are, therefore, aware that verification of hypotheses formulated on the basis of this type of study, in which the effect of causal and/or protective factors on a cohort is studied over time, has mainly a heuristic value. This type of observational/naturalistic study has in fact a value more in generating hypotheses than in being able to verify them.

Verification will be made possible through historical comparisons with other cohorts or when, in a natural way, it will be possible to observe two cohorts, one of which exposed and the other unexposed to the risk/protection factors that this study appears to suggest (for example, one supported with food and the other without, one with the possibility of returning home while the other without this).

## Conclusion

It is essential to recognize that war and war-related traumas affect people in a number of ways and that several factors can be effective in protecting individuals and their functioning. Our findings confirm that when physical conditions improve, psychological symptoms may also improve. In the sample studied, psychological factors such as the hope of returning to their own land and, thus, the possibility of maintaining ethnic cohesion may have played a role.

Future studies are needed, to be carried out with a correct methodology and adequate resources to identify protective factors, so that intervention plans can be implemented.

## Ethics Statement

Written informed consent was obtained from each participant in the study. Each participant was identified with a code number without collecting any data leading to identification of individuals. The ethics committee of the Azienda Mista Ospedaliero Universitaria, Cagliari, Italy approved the study, which had also been approved by the local camp authorities. The research was conducted according to the Helsinki Declaration.

## Author Contributions

MC, FO, and DB conceived the study, organized the collection of data, collaborated in the statistical analysis, discussed the results, and drafted the manuscript. MM, DM, and LM participated in the design of the study, performed the statistical analysis, discussed the results, and helped to draft the manuscript. MP, EP, EP-F, FS, and AP participated in the design of the study, discussed the results, and helped to draft the manuscript. All authors read and approved the final manuscript.

## Conflict of Interest Statement

The authors declare that the research was conducted in the absence of any commercial or financial relationships that could be construed as a potential conflict of interest.
